# Time and Antigen-Stimulation History Influence Memory CD8 T Cell Bystander Responses

**DOI:** 10.3389/fimmu.2017.00634

**Published:** 2017-06-08

**Authors:** Matthew D. Martin, Qiang Shan, Hai-Hui Xue, Vladimir P. Badovinac

**Affiliations:** ^1^Department of Pathology, Carver College of Medicine, University of Iowa, Iowa City, IA, United States; ^2^Department of Microbiology, Carver College of Medicine, University of Iowa, Iowa City, IA, United States; ^3^Interdisciplinary Program in Immunology, Carver College of Medicine, University of Iowa, Iowa City, IA, United States

**Keywords:** CD8 T cells, memory, bystander responses, cytokines, time-dependent functions, antigen-exposure history

## Abstract

Memory CD8 T cells can be activated and induced to produce cytokines and increase stores of cytolytic proteins not only in response to cognate antigen (Ag) but also in response to inflammatory cytokines (bystander responses). Importantly, bystander memory CD8 T cell functions have been shown to be dependent upon memory CD8 T cell fitness, since exhausted CD8 T cells have diminished capacity to respond to inflammatory cues. While it is known that memory CD8 T cell functional abilities, including ability to produce cytokines in response to cognate Ag, change with time after initial Ag encounter and upon multiple Ag stimulations (e.g., primary vs. tertiary CD8 T cell responses), it is unknown if bystander memory CD8 T cell responses are influenced by time or by Ag-exposure history. Here, we examined time and Ag-stimulation history-dependent alterations in virus-specific memory CD8 T cell bystander functions in response to inflammatory cytokines and unrelated bacterial infection. We found that expression of cytokine receptors and ability to produce IFN-γ following heterologous infection or incubation with inflammatory cytokines decreases with time following initial Ag encounter and increases with additional Ag encounters, suggesting that the ability to sense inflammation and respond with bystander cytokine production is dependent on age and Ag-stimulation history of memory CD8 T cells. These data shed further light on the regulation of memory CD8 T cell effector functions and have important implications for the development of vaccines designed to elicit protective memory CD8 T cells.

## Introduction

CD8 T cell effector functions, including cytokine secretion and targeted delivery of cytolytic molecules to infected target cells, are critical for the clearance of invading intracellular bacterial, viral, and parasitic infections (1). Following infection or vaccination, low numbers of naïve CD8 T cells recognizing cognate antigen (Ag) proliferate and give rise to an effector CD8 T cell population, which eventually undergoes contraction and forms a stable memory CD8 T cell pool (2–5). Because memory CD8 T cells are capable of providing the host with increased protection against reinfection, understanding how CD8 T cell effector functions that effect clearance of invading pathogens are regulated may aid in the development of vaccination strategies designed to elicit protective memory CD8 T cells.

While memory CD8 T cells are maintained in stable numbers with time after infection, recent work has shown that phenotype and function of primary (1°) memory CD8 T cells changes with time after initial Ag encounter (6–8). Furthermore, the properties of memory CD8 T cells that have encountered Ag multiple times have been shown to change sequentially with each additional Ag encounter (9–12). CD8 T cell activation leading to the release of cytokines and delivery of cytolytic molecules is most often thought of as being driven by cognate Ag recognition, and work describing time and Ag-stimulation history-dependent changes in CD8 T cell cytokine-producing abilities have examined Ag-dependent CD8 T cell activation. However, memory CD8 T cell activation leading to IFN-γ production and increased stores of cytolytic molecules has also been shown to be driven in an Ag-independent manner (13). At present it is unknown if Ag-independent activation, otherwise known as bystander responses, of memory CD8 T cells are dependent upon time after initial Ag encounter or Ag-stimulation history.

In mice, *in vivo* models to examine bystander CD8 T cell responses have examined activation of memory CD8 T cells following infection of mice with pathogens that do not express cognate Ag, in most cases *Listeria monocytogenes* (LM), so that mounted effector responses occur in an Ag-independent, bystander manner (14–16). Subsequent research has shown that bystander CD8 T cell responses are driven by inflammatory cytokines, and while combinations of IL-12 and IL-18 were capable of driving the most robust responses, a systematic analysis of over 1,800 combinations of inflammatory cytokines recently indicated that many different combinations of inflammatory cytokines are capable of driving bystander responses (17–21). Studies in IFN-γ-deficient mice have suggested that bystander CD8 T cell responses have the capability of providing the host with a protective benefit, although it is less clear if there is any protective benefit in immunocompetent hosts (14, 16, 22–24). While bystander CD8 T cell responses were originally described in mice, Ag-experienced human CD8 T cells have also been shown to be capable of bystander activation following stimulation with inflammatory cytokines or in response to non-related infection (25–28). Importantly, a recent article showed that exhausted CD8 T cells down-regulate expression of IL-18Rα and become unresponsive during heterologous infection or when cultured with inflammatory cytokines (29), suggesting that memory CD8 T cell bystander functions are dependent upon the overall fitness of memory CD8 T cells. Because memory CD8 T cells of different ages relative to initial infection and of different Ag-stimulation histories possess different functional abilities, memory CD8 T cell bystander responses may be dependent upon time after Ag encounter and number of Ag encounters.

Here, we used *in vivo* models to elicit bystander responses by virus-specific memory CD8 T cells in response to LM not expressing cognate Ag, as well as *in vitro* models of inflammatory cytokine-driven memory CD8 T cell IFN-γ production to examine the effects of time after initial Ag encounter and number of Ag encounters on memory CD8 T cell bystander functions. We found that memory CD8 T cell ability to sense inflammation and respond with bystander cytokine production increases with additional Ag stimulations, but decreases with time following last Ag encounter. These data shed light on the regulation of CD8 T cell effector functions and have important implications for the protective abilities of memory CD8 T cells following infection with diverse pathogens.

## Materials and Methods

### Mice, Infections, and Generation of Memory CD8 T Cells

Inbred female C57Bl/6 mice, TCR Tg P14 mice, and IFN-γ knockout (GKO) mice were bred at the University of Iowa. Outbred NIH Swiss mice were purchased from Charles River Laboratories. All mice were used at 6–10 weeks of age and housed at the University of Iowa at appropriate biosafety levels. Mice were handled and treated in accordance with the University of Iowa Institutional Animal Care and Use Committee.

**Figure 1:**
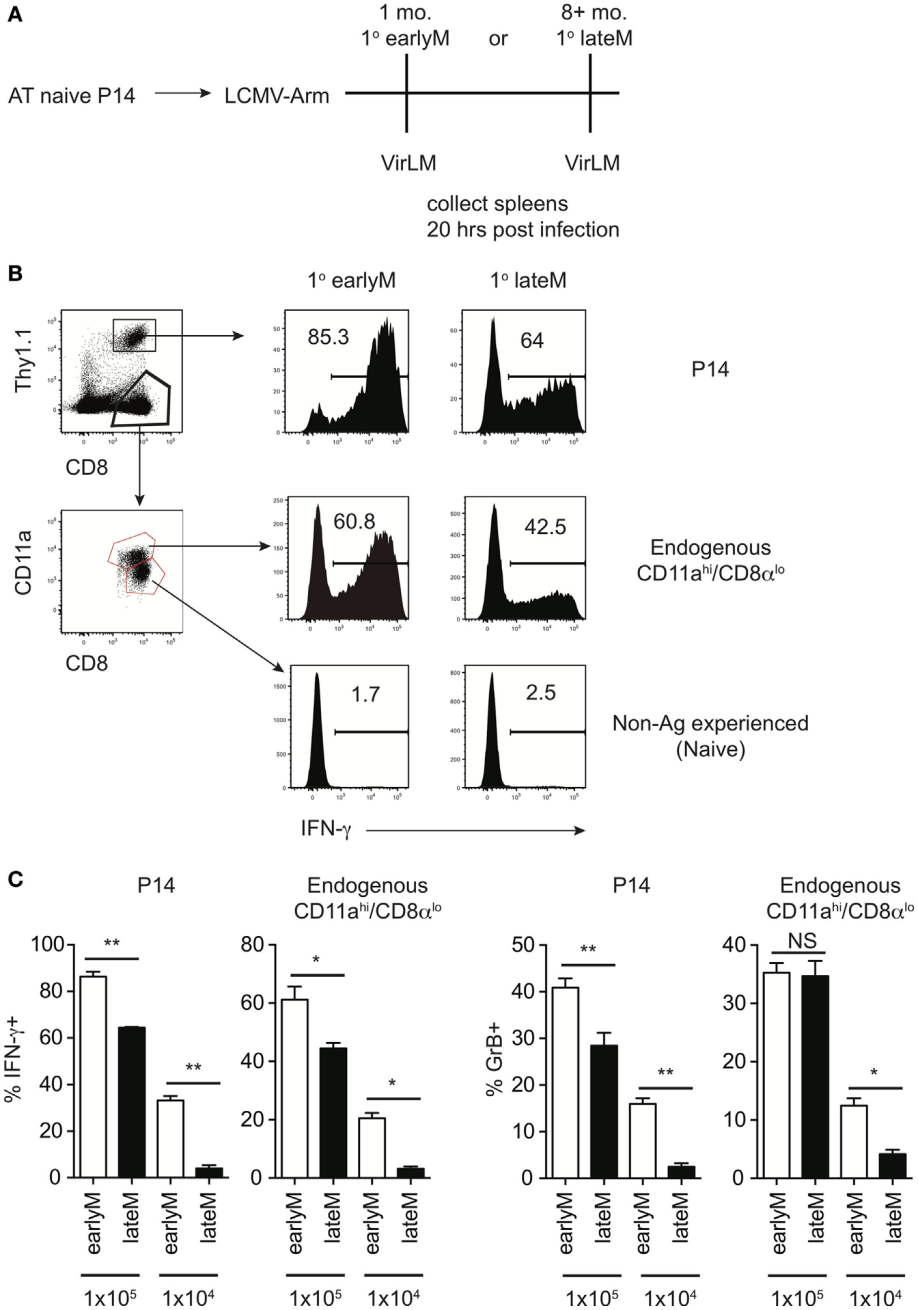
Bystander memory CD8 T cell responses decrease with time after initial antigen encounter. **(A)** Experimental Design. Mice received adoptive transfer of naïve P14 cells and were infected with LCMV-Armstrong. Either 30 days (earlyM) or >8 months (lateM) after LCMV infection, mice were infected with Vir LM. Analysis was performed 20 h following Vir LM infection. **(B)** Representative histograms of bystander IFN-γ production by earlyM (left) or lateM (right) P14 cells (top), endogenous CD11a^hi^/CD8α^lo^ Ag-experienced cells (middle), or endogenous CD11a^lo^/CD8α^hi^ naïve cells (bottom). **(C)** Summary bar graphs of the percentage of earlyM and lateM P14 cells or endogenous Ag-experienced cells producing IFN-γ or GrB 20 h after Vir LM infection of the indicated dose. *n* = 5 mice/group. Representative data from 1 of 3 independent experiments. Error bars represent mean ± SEM. Unpaired *t*-test; NS, not significant, *p < 0.05, **p < 0.01.

All LCMV Armstrong infections were performed intraperitoneally with 2 × 10^5^ plaque forming units per mouse. All LM infections (Figures 1 and 7; Figures S1–S3 in Supplementary Material) were performed intravenously with virulent LM strain 10403S at approximately 1 × 10^4^, 1 × 10^5^, or 1 × 10^6^ colony forming units (CFU) per mouse or at the dose indicated in the figure legend.

1° memory P14 cells were generated by adoptively transferring 5 × 10^3^ P14 cells obtained from peripheral blood of naïve P14 mice (Thy1.1/1.1 or Thy1.1/1.2) into naïve C57Bl/6 recipients (Thy1.2/1.2) followed by infection with LCMV. Tertiary (3°) memory CD8 T cells were generated by serial adoptive transfer of naïve, 1° memory, and secondary (2°) memory P14 cells. To generate 2° memory P14 cells, 2 × 10^4^ P14 cells from spleens of mice containing 1° memory P14 cells greater than 30 days following LCMV infection were transferred into new naïve C57Bl/6 mice, followed by LCMV infection. To generate 3° memory P14 cells, 5 × 10^4^ P14 cells from spleens of mice containing 2° memory P14 cells greater than 30 days following LCMV infection were transferred into new naïve C57Bl/6 mice, followed by LCMV infection. EarlyM P14 cells were analyzed ~1 month following infection and lateM P14 cells were analyzed >8 months following infection.

For challenge experiments in GKO mice, naïve GKO mice received or did not receive adoptive transfer of 4 × 10^5^ 1° earlyM or lateM, or 1° or 3° earlyM P14 cells that were purified with anti-PE magnetic bead sorting (>95% purity) using standard AutoMacs protocols (Miltenyi Biotec). Mice were challenged with 1 × 10^4^ CFU of virulent LM, and colonies present in the liver were determined 2 days after infection.

### Flow Cytometry and Intracellular Cytokine Staining

Spleens were collected and tissue was processed into single-cell suspension. Surface staining was conducted by incubating splenocytes with appropriate antibody cocktails for 20 min at 4°C. Endogenous memory and naïve CD8 T cells were detected based upon surface staining with anti-CD8 (clone 53-6.7, eBioscience) and anti-CD11a (clone M17/4, eBioscience) as previously described (30). P14 cells were detected based upon surface staining with anti-CD8 and anti-Thy1.1 (clone His51, eBioscience), and in some instances 1° earlyM and lateM or 1° and 3° memory P14 cells were distinguished from one another based upon additional surface staining with anti-Thy1.2 (clone 53-2.1, eBioscience). Intracellular cytokine staining was performed using anti-IFN-γ (clone XMG1.2, eBioscience) or anti-granzymeB (anti-GrB; clone GB12, Invitrogen). Flow cytometry data was acquired using FACSCanto (BD Biosciences, San Jose, CA, USA) and analyzed using FloJo software (Tree Star Inc., Ashland, OR, USA).

### Detection of *In Vitro* and *In Vivo* Bystander Memory CD8 T Cell Responses

For *in vitro* bystander responses, splenocytes were isolated from mice containing memory P14 cells and were incubated for 4 h at 37°C with rIL-12 and IL-18 or IL-12 and TNF-α or IL-18 and IFN-β (R&D Systems) (10 ng/mL each). Thy disparate 1° earlyM and lateM or 1° and 3° memory P14 cells were mixed together prior to incubation. Cells were incubated for 1 additional hour in the presence of Brefeldin A (BFA) before surface and intracellular staining.

For *in vivo* bystander responses, mice containing 1° earlyM or lateM P14 cells generated in response to LCMV infection were infected with the indicated doses of LM. 20 h following LM infection (unless otherwise stated), splenocytes were isolated and incubated with BFA for 1 h before surface and intracellular staining.

### Quantitative RT-PCR and Western Blot

Spleens of mice containing earlyM or lateM P14 cells were collected and tissue was processed into single-cell suspension. Cells were surface stained with anti-CD8 and anti-Thy1.1 and sorted using a BD FACSAria II (BD Biosciences, San Jose, CA, USA).

For quantitative RT-PCR, total RNA was reverse-transcribed using a QuantiTech Reverse Transcription Kit (Qiagen). The resulting cDNA was analyzed for expression of *Il12rb1, Il12rb2, Il18r1*, or *Il18rap* by quantitative PCR using SYBR Advantage qPCR premix (Clontech) on an ABI 7300 Real Time PCR System (Applied Biosystems). Relative gene expression levels in each sample were normalized to that of a housekeeping gene, hypoxanthine phosphoribosyltransferase 1 (*Hprt1*).

The primers used in quantitative RT-PCR were as follows:

*Il12rb1*:5′-TGTGTTTCTGAGCGTGGACA and 3′-CCTGAGGCGCCTAGCTG
*Il12rb2*:5′-GTGTCTGCAGCCAACTCAAA and 3′-AGGCTGCCAGGTCACTAGAA
*Il18r1*:5′-CTCCCTGTCTGTTGTCACAGT and 3′-GGTATCTCTTGTTTTCAGGATCGTT
*Il18rap*:5′-AGAAGGCGAATAGTGGTGGC and 3′-TGGTGGCACAAGCTGAATGAT

For Western blot, cells sorted from three mice were combined and lysed, and whole-cell lysates were clarified by centrifugation and resolved by SDS-PAGE (BIO-RAD). Protein was detected using antibodies against IL-12Rβ2 (ab67365, Abcam) and ACTB (ab8226, Abcam).

## Results

### Bystander Memory CD8 T Cell Responses to Non-Related Infection Decrease with Time after Initial Ag Encounter

Before addressing if bystander functions of memory CD8 T cells are influenced by time following initial Ag encounter, we wanted to establish a model that would allow us to examine differences in bystander responses for memory cells of differing functional abilities. We began by using a well-established *in vivo* model to elicit bystander memory CD8 T cell responses in the absence of cognate Ag, adoptively transferring naïve TCR-transgenic P14 cells specific for GP33 LCMV-derived epitope into recipient mice followed by LCMV-Arm infection, and at a memory time point infecting mice with LM, which does not express Ag recognized by the memory P14 cells. Bystander memory CD8 T cell responses were dependent upon the dose of LM used, as a stepwise increase in the percentage of P14 cells producing IFN-γ was observed upon infection with 1 × 10^4^, 1 × 10^5^, and 1 × 10^6^ CFUs of LM (Figure S1A in Supplementary Material). To determine kinetics of IFN-γ production, mice containing memory P14 cells were infected with 1 × 10^5^ CFUs of LM, and IFN-γ production by P14 cells was assessed 1, 5, 24, and 48 h after infection. The highest percentage of P14 cells producing IFN-γ was detected 20 h after infection (Figure S1B in Supplementary Material). Based on these data, we infected mice with 1 × 10^4^ or 1 × 10^5^ CFUs of LM for further studies, and bystander CD8 T cell responses were examined 20 h after LM infection.

Of note, the percentage of memory CD8 T cells producing IFN-γ following infection with 1 × 10^5^ CFUs LM was different in the two experiments (Figure S1A in Supplementary Material 1 × 10^5^ CFU and S1B 20 h). This could have been due to experiment to experiment variability, however, the age of memory CD8 T cells relative to initial Ag encounter was different between the two experiments, which suggested the possibility that memory CD8 T cell bystander functions may be dependent upon time after initial Ag encounter. To explore this further, we generated mice containing P14 memory cells and administered LM infection either 1 month (earlyM) or >8 months (lateM) after primary LCMV infection (Figure 1A). Based on a surrogate activation marker strategy to detect Ag-experienced CD8 T cells (30), only Ag-experienced CD8 T cells were able to produce IFN-γ in response to non-related LM infection, as CD11a^lo^/CD8α^hi^ CD8 T cells did not produce IFN-γ (Figure 1B). Interestingly, the percentage of both memory transgenic P14 cells and endogenous CD11a^hi^/CD8α^lo^ memory CD8 T cells that produced IFN-γ or increased stores of GrB was greater in earlyM compared to lateM mice (Figures 1B,C). Similar results for bystander IFN-γ production by endogenous Ag-experienced memory CD8 T cells were seen in outbred NIH Swiss mice (Figures S2A,B in Supplementary Material). Taken together, these data suggest that the ability of memory CD8 T cells to execute bystander responses following non-related infection decreases with time after initial Ag encounter.

### Memory CD8 T Cell Ability to Sense and Respond to Inflammation with IFN-γ Production Decreases with Time after Initial Ag Encounter

**Figure 2:**
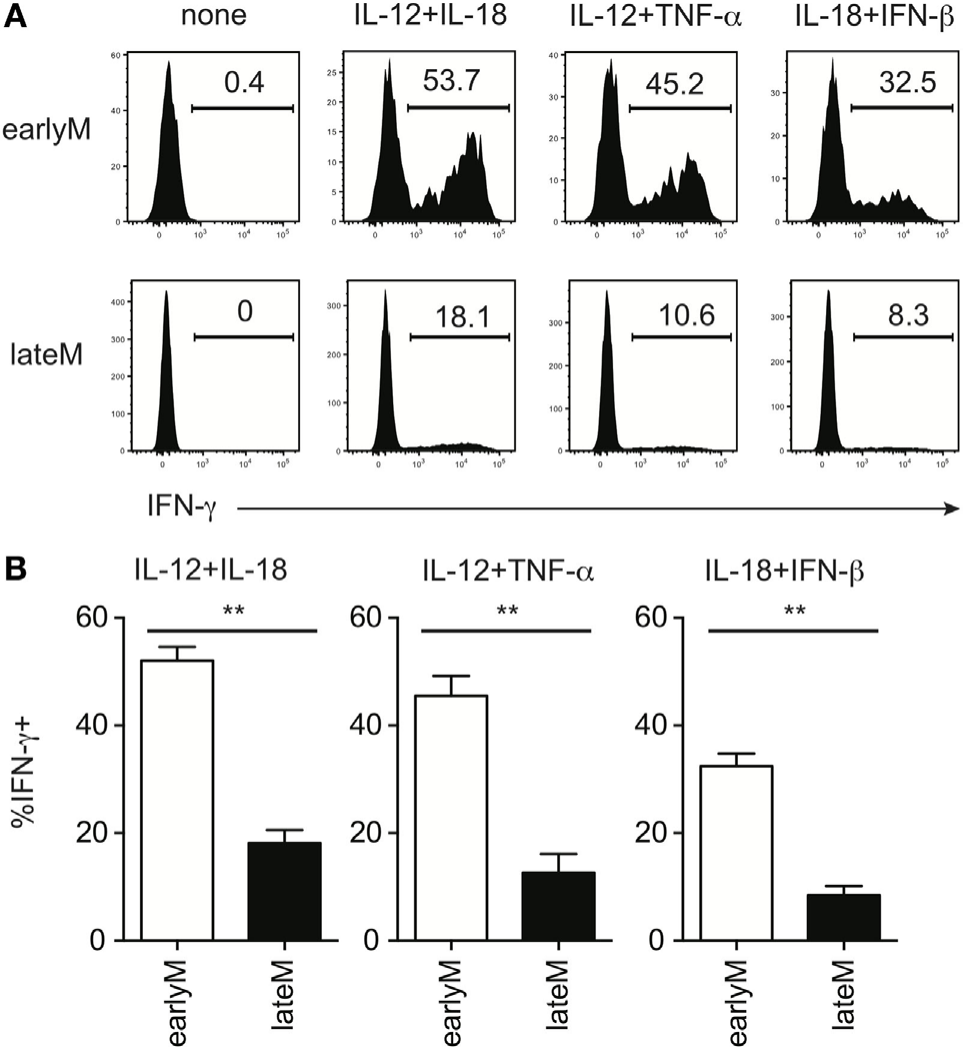
Memory CD8 T cell IFN-γ production in response to inflammatory cytokines decreases with time after initial antigen encounter. Mice received adoptive transfer of naïve P14 cells and were infected with LCMV-Armstrong. Either 30 days (earlyM) or >8 months (lateM) after LCMV infection, splenocytes were harvested; mixed together; and incubated for 4 h with 10 ng/mL each of rIL-12 and IL-18, IL-12 and TNF-α, or IL-18 and IFN-β in the absence of Brefeldin A and for 1 additional hour in the presence of Brefeldin A. **(A)** Representative histograms of IFN-γ production by earlyM (top) or lateM (bottom) P14 cells incubated with the indicated combinations of inflammatory cytokines. **(B)** Summary bar graphs of the percentage of earlyM or lateM P14 cells producing IFN-γ following incubation with the indicated combinations of inflammatory cytokines. *n* = 3 mice/group. Representative data from 1 of >3 independent experiments. Error bars represent mean ± SEM. Unpaired *t*-test; **p < 0.01.

Bystander memory CD8 T cell responses are driven by recognition of inflammatory cytokines generated in response to infection by cytokine receptors present on the memory cells (18, 21, 29). In the context of LM infection, the primary inflammatory cytokines responsible for driving bystander responses are likely IL-12 and IL-18 (14, 31–33). However, Ag-experienced CD8 T cells can respond to a wide range of inflammatory cytokine combinations with IFN-γ production (21). To examine if inflammatory cytokine-driven memory CD8 T cell bystander responses were dependent upon the age of the memory CD8 T cells relative to initial Ag encounter, we mixed Thy disparate earlyM and lateM P14 cells, which normalized the incubation environment and allowed us to examine cell intrinsic differences between earlyM and lateM cells, with combinations of inflammatory cytokines and examined IFN-γ production. In response to incubation with 3 different combinations of inflammatory cytokines, at least twice as many earlyM compared to lateM P14 cells produced IFN-γ (Figures 2A,B), suggesting that memory CD8 T cell ability to produce IFN-γ in response to inflammatory cytokines decreases with time.

**Figure 3:**
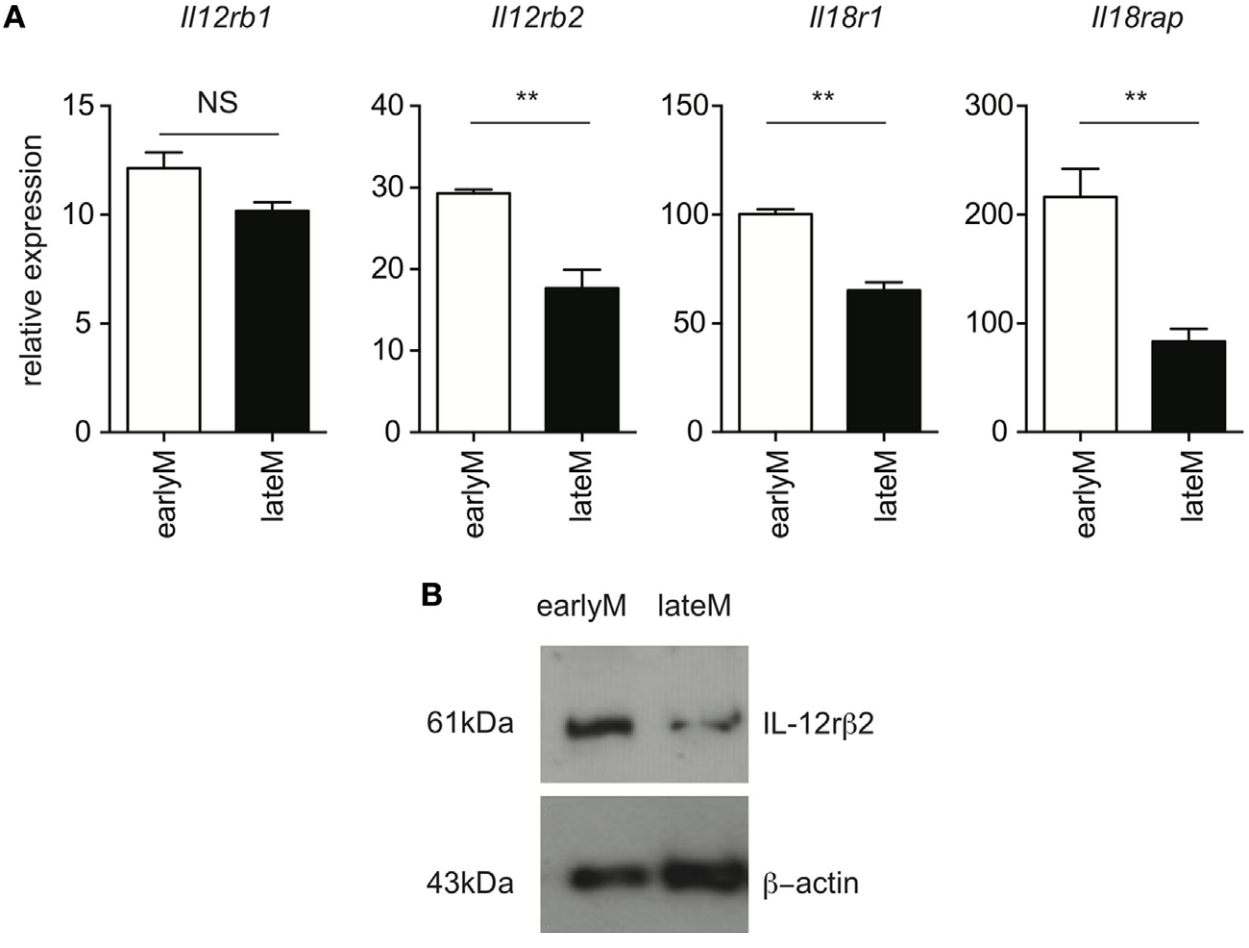
Expression of components of the IL-12 and IL-18 receptors decreases with time after initial antigen encounter. Mice received adoptive transfer of naïve P14 cells and were infected with LCMV-Armstrong. Either 30 days (earlyM) or >8 months (lateM) after LCMV infection, splenocytes were sorted. **(A)** Summary bar graphs of relative expression of *Il12rb*1, *Il12rb2, Il18r1*, and *Il18rap* based on qRT-PCR for earlyM or lateM P14 cells. *n* = 3 mice per group. Representative data from 1 of 2 independent experiments. Error bars represent mean ± SEM. Unpaired *t*-test; NS, not significant, **p < 0.01. **(B)** Western blot image of expression of IL-12rβ2 for earlyM and lateM P14 cells. P14 cells were sorted from three separate mice and combined. Representative image from one of two independent experiments with similar results.

Ability to produce IFN-γ in response to inflammatory cytokines is based at least in part upon the ability to sense inflammatory cytokines in the environment through cytokine receptors. Recently, it was shown that exhausted CD8 T cells downregulate expression of cytokine receptors (29), suggesting that cytokine receptor expression on memory CD8 T cells is dependent upon their overall fitness. We were unable to reliably detect expression of IL-12 and IL-18 receptor components by flow cytometry using commercially available antibodies. Therefore, to determine whether qualitative differences, including differences in expression of cytokine receptors, between earlyM and lateM CD8 T cells could explain differences in ability to execute bystander functions, we sorted earlyM and lateM P14 cells and determined expression of components of IL-12 and IL-18 receptors by qRT-PCR and Western blot. mRNA expression of components of the IL-12 and IL-18 receptors (Figure 3A), and protein expression of IL-12rβ2 (Figure 3B) was lower on lateM compared to earlyM P14 cells, suggesting that expression of IL-12 and IL-18 receptors on memory CD8 T cells decreases with time after Ag encounter. Taken together, the data presented in Figures 1–3 suggest that the ability of memory CD8 T cells to respond in a bystander manner decreases with time after initial Ag encounter due to a decreased ability to sense and respond to inflammatory cytokines.

### Cytokine Receptor Expression and Ability to Produce IFN-γ in Response to Inflammatory Cytokines Increases with Repetitive Ag Stimulations

**Figure 4:**
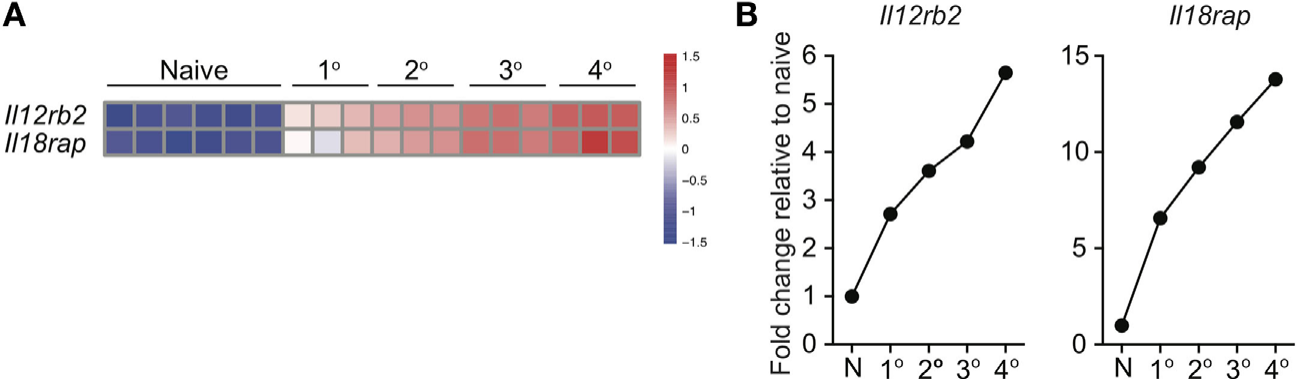
Expression of IL-12 and IL-18 receptor components increases with additional antigen encounters. 1°, 2°, 3°, and 4° memory OT-I cells were generated and microarray analysis was performed as described by Wirth et al. Relative gene expression was determined from the microarray date deposit available in the Gene Expression Omnibus database (http://www.ncbi.nlm.nih.gov/gds) under the accession number GSE21360. **(A)** Heatmap of expression of *Il12rb2* and *Il18rap* for naïve, 1°, 2°, 3°, and 4° memory OT-I cells. **(B)** Fold change in expression of *Il12rb2* and *Il18rap* for 1°, 2°, 3°, and 4° memory OT-I cells compared to expression for naïve OT-I cells.

**Figure 5:**
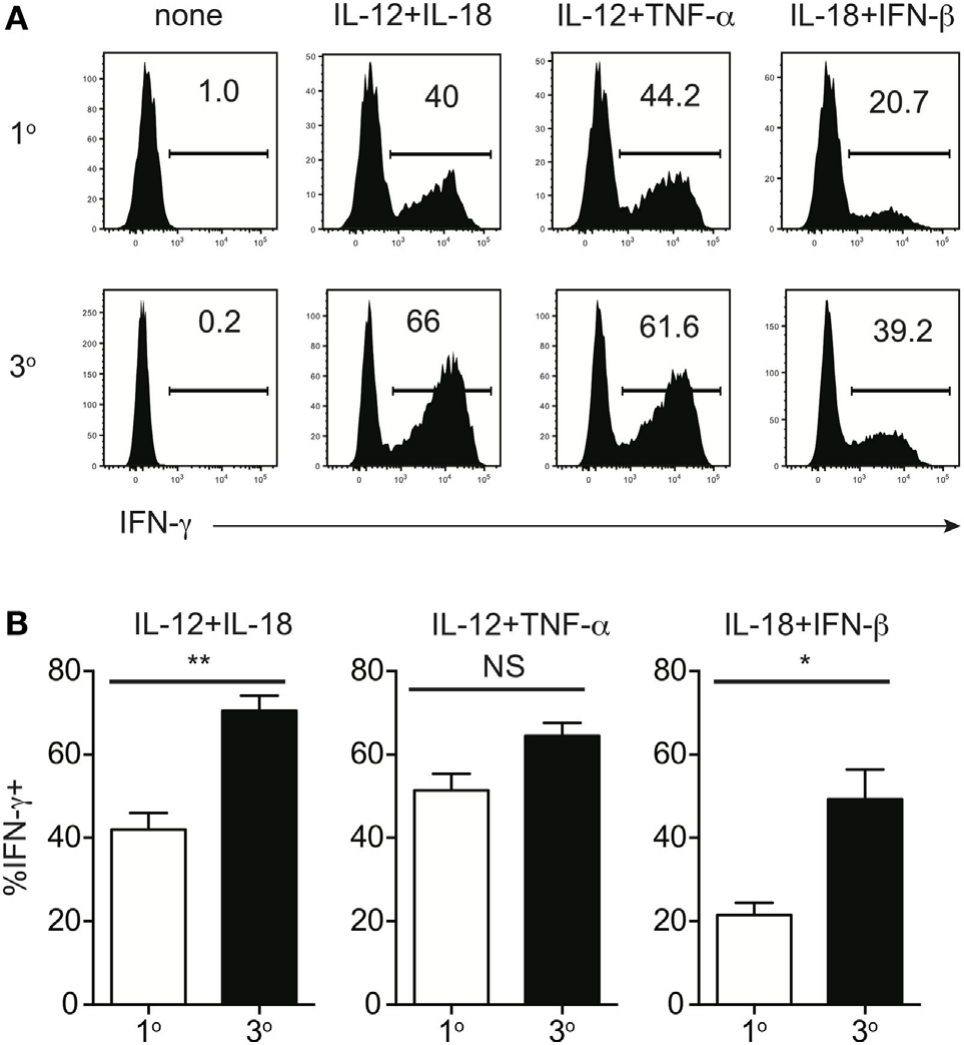
Memory CD8 T cell IFN-γ production in response to inflammatory cytokines increases with additional antigen encounters. Mice received adoptive transfer of naïve or 2° memory P14 cells and were infected with LCMV-Armstrong. 30 days after LCMV infection, splenocytes were harvested; mixed together; and incubated for 4 h with 10 ng/mL each of rIL-12 and IL-18, IL-12 and TNF-α, or IL-18 and IFN-β in the absence of Brefeldin A and for 1 additional hour in the presence of Brefeldin A. **(A)** Representative histograms of IFN-γ production by 1° (top) or 3° (bottom) memory P14 cells incubated with the indicated combinations of inflammatory cytokines. **(B)** Summary bar graphs of the percentage of 1° or 3° memory P14 cells producing IFN-γ following incubation with the indicated combinations of inflammatory cytokines. *n* = 3 mice/group. Representative data from 1 of >3 independent experiments. Error bars represent mean ± SEM. Unpaired *t*-test; NS, not significant, *p < 0.05, **p < 0.01.

Because of prime-boost vaccination regimens and repeated exposure to the same infection, memory CD8 T cells often encounter cognate Ag multiple times. Research has shown that functions, including Ag-dependent cytokine production, of memory CD8 T cells that have encountered Ag multiple times (primary to quaternary—1° to 4°) changes with each subsequent Ag encounter (9–12). Microarray data previously generated in our laboratory (11) demonstrated that expression of components of the IL-12 and IL-18 receptors increases in a stepwise-manner among 1°, 2°, 3°, and 4° memory CD8 T cells analyzed at the same day after the last Ag encounter (Figures 4A,B), suggesting that memory CD8 T cell bystander functions also may be dependent upon Ag-stimulation history. To examine this further, we generated 1° and 3° memory P14 cells by serial adoptive transfer followed by LCMV infection, and incubated 1° and 3° memory cells with inflammatory cytokine combinations followed by intracellular cytokine staining to determine IFN-γ production. While differences in cytokine receptor expression were further magnified with each additional Ag encounter, we chose to examine differences between 1° and 3° memory CD8 T cells because differences in cytokine receptor expression between 1° and 3° memory cells mice were robust, and mice containing 3° memory cells require significantly less time to generate than mice containing 4° memory cells. As was suggested by increased expression of cytokine receptor components in 3° compared to 1° memory cells, a greater percentage of 3° compared to 1° memory P14 cells produced IFN-γ in response to rIL-12 and IL-18 and IL-18 and IFN-β (Figures 5A,B), suggesting that the ability of memory CD8 T cells to sense and respond to inflammatory cytokines with IFN-γ production in a bystander manner increases with additional Ag encounters.

### Bystander IFN-γ Production in Response to Inflammatory Cytokines Decreases with Time after Initial Ag Encounter for 3° Memory Cells

**Figure 6:**
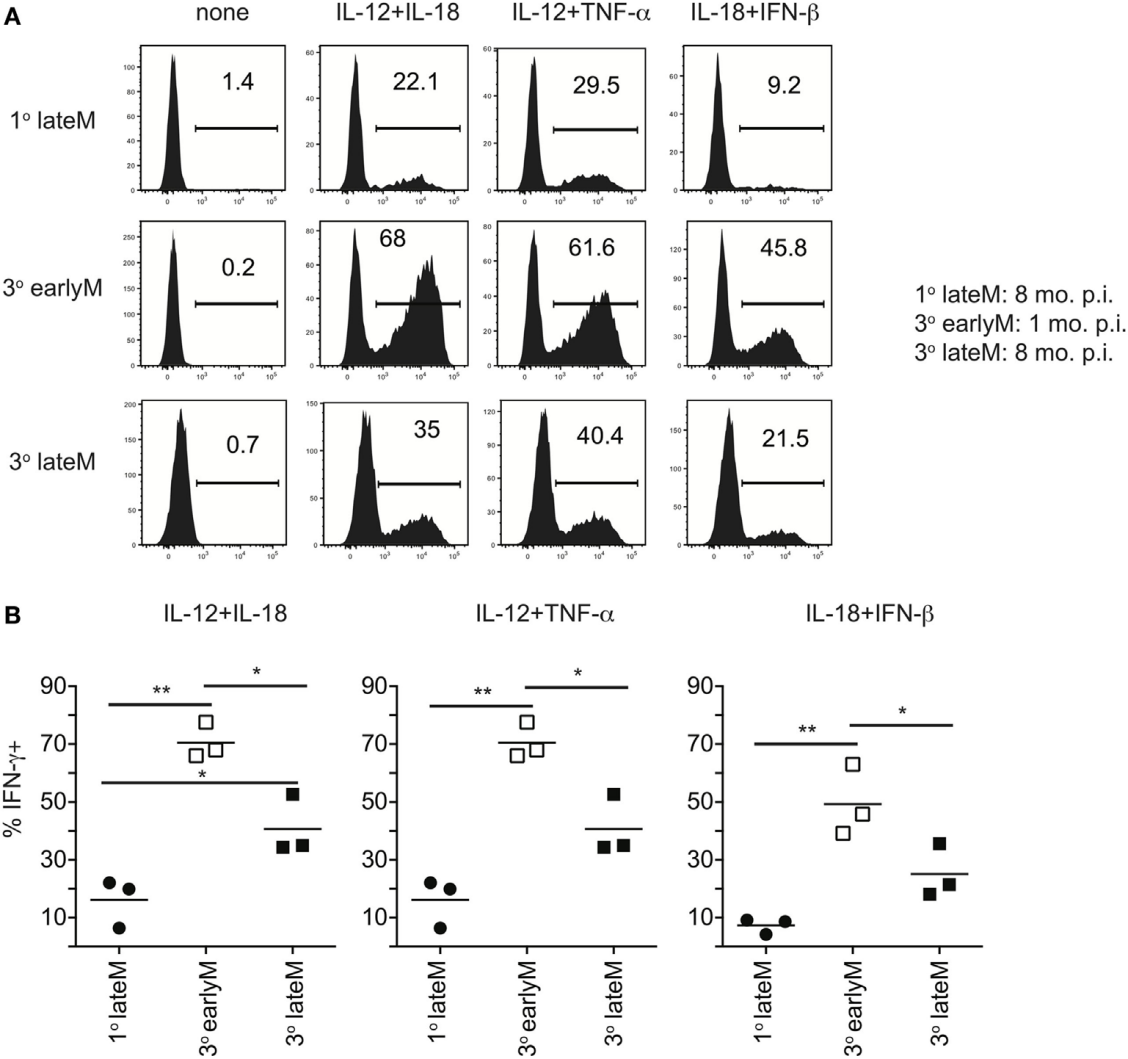
IFN-γ production in response to inflammatory cytokines decreases with time after initial antigen encounter for 3° memory CD8 T cells. Mice received adoptive transfer of naïve or 2° memory P14 cells and were infected with LCMV-Armstrong. 30 days (3° earlyM) or >8 months (1° lateM and 3° lateM) after LCMV infection, splenocytes were harvested and incubated for 4 h with 10 ng/mL each of rIL-12 and IL-18, IL-12 and TNF-α, or IL-18 and IFN-β in the absence of Brefeldin A and for 1 additional hour in the presence of Brefeldin A. **(A)** Representative histograms of IFN-γ production by 1° lateM (top), 3° earlyM (middle), or 3° lateM (bottom) P14 cells incubated with the indicated combinations of inflammatory cytokines. **(B)** Summary graphs of the percentage of 1° lateM, 3° earlyM, or 3° lateM P14 cells producing IFN-γ following incubation with the indicated combinations of inflammatory cytokines. *n* = 3 mice/group. Representative data from one of three independent experiments. Bars represent mean. ANOVA with Bonferroni posttest; *p < 0.05, **p < 0.01.

We previously showed that ability to undergo bystander activation decreases with time after initial Ag encounter for 1° memory CD8 T cells due to qualitative changes in the memory CD8 T cells that occur with time (Figures 1–3). While the question of whether qualitative changes occur with time after Ag encounter for memory CD8 T cells that have encountered Ag multiple times has remained relatively unexplored, some research has suggested that, like 1° memory CD8 T cells, the phenotype and function of memory CD8 T cells that have encountered Ag multiple times changes with time after initial Ag encounter (9–12). To determine if bystander responses of memory CD8 T cells that have encountered Ag multiple times are dependent upon time after initial Ag encounter, we incubated 3° earlyM and 1° and 3° lateM P14 cells with combinations of inflammatory cytokines and assessed IFN-γ production. As previously shown (Figure 5), a greater percentage of 3° compared to 1° memory P14 cells produced IFN-γ following incubation with inflammatory cytokine combinations (Figures 6A,B), which provides further evidence that the ability of memory CD8 T cells to produce IFN-γ in a bystander manner increases with additional Ag encounters. However, a decreased percentage of 3° lateM compared to 3° earlyM cells produced IFN-γ following incubation with all combinations of cytokines tested (Figures 6A,B), suggesting that bystander responses of memory CD8 T cells that have encountered Ag multiple times also decrease with time after Ag encounter. Taken together, the data presented in Figures 1–6 suggest that the ability of memory CD8 T cells to sense inflammation and undergo bystander activation increases for memory CD8 T cells with additional Ag encounters but decreases with time after last infection.

### Despite Differences in Bystander Functions, Time after Ag Encounter and Ag-Stimulation History Do Not Impact Protection Provided against Non-Related LM Infection

**Figure 7:**
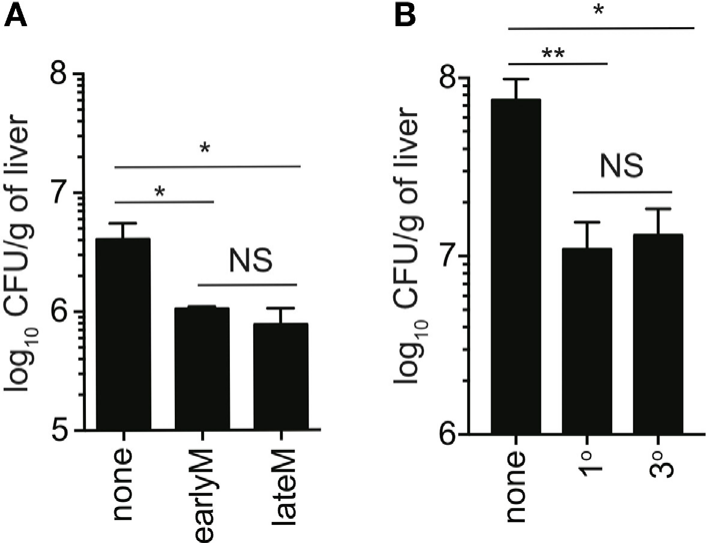
Ability to provide protection against unrelated LM infection is not influenced by time after initial antigen (Ag) encounter or Ag-encounter history. IFN-γ knockout mice either received or did not receive adoptive transfer of 400,000 1° earlyM or lateM P14 cells **(A)**, or 1° or 3° earlyM P14 cells **(B)**, and were infected with Vir LM [**(A)** 0.8 × 10^4^ CFU and **(B)** 1.3 × 10^4^ CFU]. Approximately 1.5 **(A)** to 2.5 **(B)** days after infection, livers were harvested and bacterial colony forming units were enumerated. **(A)** Summary bar graph of LM colonies detected in livers of IFN-γ knockout mice that received adoptive transfer of the indicated populations of 1° memory P14 cells. *n* = 3 mice/group. Representative data from one of three independent experiments. Bars represent mean ± SEM. ANOVA with Bonferroni posttest; NS, not significant, *p < 0.05. **(B)** Summary bar graph of LM colonies detected in livers of IFN-γ knockout mice that received adoptive transfer of the indicated populations of 1° or 3° earlyM P14 cells. *n* = 3 mice/group. Representative data from one of three independent experiments. Bars represent mean ± SEM. ANOVA with Bonferroni posttest; NS, not significant, *p < 0.05, **p < 0.01.

Bystander IFN-γ production has the potential to provide the host with a protective benefit during non-related infections. Currently, the best-studied model to test this is infection with non-related LM, and the current literature suggests that bystander responses during non-related LM infection do not provide immunocompetent hosts with any protective benefit (24). This is likely because IFN-γ produced by other immune cells is sufficient to combat the infection, and any additional IFN-γ produced by bystander activation does not appreciably add to IFN-γ produced by innate lymphocytes. However, in IFN-γ knockout mice, in which the only cells capable of producing IFN-γ are IFN-γ competent memory cells transferred into the hosts, bystander responses are capable of providing significant levels of protection (14, 16, 22–24). To determine if time and Ag-stimulation history-dependent differences in ability to undergo bystander activation impact clearance of infection with non-related pathogens, we sorted 1° earlyM and lateM or 1° and 3° earlyM cells (4 × 10^5^/each) and transferred into IFN-γ knockout mice followed by LM infection, and assessed bacterial clearance in the liver 2 days after infection. While adoptive transfer of memory cells of any type provided protection compared to mice that did not receive memory cells, no significant differences in protection provided were seen between 1° earlyM and lateM (Figure 7A) or 1° and 3° earlyM cells (Figure 7B). Additionally, no differences in bystander-mediated protection were seen between 1° earlyM, 1° lateM, and 3° memory in the spleen, another organ in which bacterial replication occurs following LM infection (Figure S3 in Supplementary Material). Thus, while the ability to sense and respond to inflammation in a bystander manner increases with additional Ag encounters and decreases with time after infection, protection observed is not influenced by the capacity of those cells to recognize inflammation cues delivered by heterologous infection.

## Discussion

In the present study, we describe how the Ag-independent bystander functions of memory CD8 T cells are impacted by time after initial Ag-encounter and Ag-stimulation history. Using *in vitro* models of exposure to inflammatory cytokines and well-established *in vivo* models of bystander infection with non-related LM, we compared bystander IFN-γ production of memory CD8 T cells early compared to late after initial Ag encounter and of 1° compared to 3° memory CD8 T cells. Our results show that the ability of memory CD8 T cells to respond to inflammation in a bystander manner decreases with time after initial Ag encounter regardless of number of Ag encounters, but increases with additional Ag encounters. These differences are likely due to qualitative changes in memory CD8 T cells of different ages relative to initial Ag encounter and of different Ag-stimulation histories that lead to differential ability to sense inflammation, as we showed that expression of components of the IL-12 and IL-18 receptors decrease with time after initial Ag encounter and increase with repetitive recognition of cognate Ag.

Memory CD8 T cell ability to produce IFN-γ and TNF-α in response to cognate Ag is maintained while ability to produce IL-2 increases with time following initial Ag encounter (7, 8). Likewise, ability to produce IFN-γ and TNF-α in response to cognate Ag is similar in 1° memory CD8 T cells and memory cells that have encountered Ag multiple times while ability to produce IL-2 decreases and intracellular stores of granzymes increase with additional Ag encounters (9–11). However, we have shown that ability to produce cytokines non-specifically in response to inflammation decreases with time after initial Ag encounter and increases with additional Ag encounters. That Ag-driven CD8 T cell responses are maintained while inflammation-driven bystander responses are time and Ag-stimulation history dependent suggests interesting regulation of effector functions of memory CD8 T cells. Our data indicate that with time after initial Ag encounter, effector responses are more tightly regulated and memory CD8 T cells become trained to only respond to infections displaying cognate Ag, while this restraint is less strict for memory CD8 T cells upon additional Ag encounters.

The memory CD8 T cell pool that is generated following contraction is comprised of a heterogeneous pool of cells with differing functions (34). Ruiz et al. (35) showed that memory CD8 T cells that express NK1.1 have an enhanced ability to respond following heterologous infection, and are more protective than NK1.1 non-expressing memory cells, suggesting that subsets of memory CD8 T cells may differ in their ability to perform bystander functions. We found, based on expression of CD62L (36, 37) or CX3CR1 (38–40) to differentiate T_em_ from T_cm_ cells, that both primary T_em_ and T_cm_ cells were able to produce IFN-γ in response to inflammatory cytokines, but that a higher percentage of T_em_ compared to T_cm_ cells were able to respond (unpublished data). Therefore, changes in the subset composition that occur with time after infection (7, 8) may contribute to differences in bystander functions of *early*M and *late*M. Furthermore, a body of literature has documented changes in the function of the immune system upon aging (41). While the literature suggests that *in vitro* bystander responses elicited by most combinations of inflammatory cytokines are directed by CD8 T cell independently of accessory cells, some responses (primarily those driven by IL-15) require the help of accessory cells (21), the function of which may be different in mice of differing ages. However, for our *in vitro* bystander assays, we coincubated *early*M and *late*M cells, so the accessory cells present in culture were the same for both *early*M and *late*M cells, and differences in IFN-γ elicited by inflammatory cytokines were truly CD8 T cell intrinsic.

Infection with pathogens expressing cognate Ag comes in the context of not only cognate Ag, but also inflammation. We previously showed that both Ag and inflammation are important for driving memory CD8 T cell-derived effector functions in response to infection displaying cognate Ag (24). Our current description of time and Ag-stimulation history dependent differences in ability to sense inflammation suggests that the relative importance of inflammation in driving CD8 T cell effector functions following infection with pathogens displaying cognate Ag may differ for memory CD8 T cells of differing ages relative to initial Ag encounter and for memory CD8 T cells that have encounter Ag multiple times. In addition to effector cytokine production, inflammatory cytokines also impact memory CD8 T cell migration and proliferation. Our own microarray data (7, 11) and that of others (8) has shown that expression of cytokine receptors in addition to components of the IL-12 and IL-18 receptors, including expression of *Il2rβ* that comprises part of the IL-15 receptor, differ between memory CD8 T cells of different ages relative to initial Ag encounter and for memory CD8 T cells that have encountered Ag multiple times. A recent article by Richer et al. showed that IL-15 signaling, which was induced upon type I IFN exposure, induced memory CD8 T cell cycle progression, resulting in greater memory CD8 T cell proliferation following Ag exposure (42). Nolz et al. found that IL-15 signaling on memory CD8 T cells promotes core 2 O-glycan synthesis, which regulates trafficking of CD8 T cells to inflamed tissues (43). Therefore, it would be interesting to examine whether qualitative differences in memory CD8 T cells of different ages relative to initial Ag encounter and of different Ag exposure histories lead to differences not only in bystander function but also to differences in ability to home to inflamed tissues or to prepare for division following Ag encounter.

Many have questioned the physiological role of bystander CD8 T cell responses. On one hand, bystander cytokine production in the context of non-related infection could lead to faster clearance of diverse pathogens, providing the host with a protective benefit. On the other hand, bystander CD8 T cell responses could be detrimental to the host in the context of autoimmune diseases or graft vs. host disease. Experimentally, the most developed model used to explore the relevance of bystander CD8 T cell responses is the clearance of non-related LM infection (14, 16, 22, 24). Using this model, it has been found that bystander responses by memory CD8 T cells adoptively transferred into IFN-γ-deficient mice, in which the only cells capable of producing IFN-γ are adoptively transferred cells producing cytokines in a bystander manner, provide significant levels of protection against LM infection. However, our recent work has suggested that bystander CD8 T cell responses in immunocompetent hosts, where abundant amounts of IFN-γ are produced in response to infection by other immune cells in addition to cells responding in a bystander manner, do not significantly add to IFN-γ levels or provide the host with a protective benefit (24). Interestingly, in the current study we found that despite having an impact on ability to perform bystander functions, time after initial Ag encounter and number of Ag encounters did not impact protection observed against LM in IFN-γ-deficient mice. Similarly to the lack of protection provided by bystander responses against non-related LM infection in IFN-γ sufficient hosts, this could be because bystander IFN-γ produced by any transferred memory cells is sufficient to provide a level of protection, but additional levels of IFN-γ of early compared to late memory or 3° compared to 1° memory do not significantly add to IFN-γ levels or protection provided. In this regard, the LM model, which elicits the production of copious amounts of inflammatory cytokines, may not be a suitable model to determine if small, but appreciable differences in ability to produce bystander IFN-γ result in differences in ability to provide protection against non-related infection.

In conclusion, we have shown how time after initial Ag-encounter and Ag-exposure history can impact regulation of bystander CD8 T cell effector functions. While Ag-dependent effector cytokine production is maintained in memory CD8 T cells with time after initial Ag encounter and with additional Ag encounters, ability to respond in a bystander manner decreases with time after Ag encounter, a notion that should further define long-lived memory CD8 T cell function *in vivo*.

## Ethics Statement

This study was carried out in accordance with the recommendations of University of Iowa Animal Care and Use Committee. The protocol was approved by University of Iowa Animal Care and Use Committee.

## Author Notes

Some of the data presented herein were obtained at the Flow Cytometry Facility, which is a Carver College of Medicine/Holden Comprehensive Cancer Center core research facility at the University of Iowa. The Facility is funded through user fees and the generous financial support of the Carver College of Medicine, Holden Comprehensive Cancer Center, and Iowa City Veteran’s Administration Medical Center.

## Author Contributions

MM, QS, H-HX, and VB designed experiments. MM performed experiments and data analysis and wrote the manuscript. QS helped obtaining some data and analyzing some data and reviewed the final manuscript. H-HX contributed to the discussion and reviewed the final manuscript. VB contributed to writing and editing of the manuscript.

## Conflict of Interest Statement

The authors declare that the research was conducted in the absence of any commercial or financial relationships that could be construed as a potential conflict of interest.

## Supplementary Material

Figure S1 Bystander memory CD8 T cell responses occur in a dose-dependent manner and are robust 20 h after infection. **(A)** Mice received adoptive transfer of naïve P14 cells and were infected with LCMV-Armstrong. 200 days after LCMV infection, mice were infected with 1 × 10^4^, 1 × 10^5^, or 1 × 10^6^ colony forming units of Vir *Listeria monocytogenes* (LM). Analysis was performed 20 h following Vir LM infection. Representative histograms of bystander IFN-γ production by gated P14 cells following the indicated infectious dose of Vir LM. Representative data from one of two independent experiments. **(B)** Mice received adoptive transfer of naïve P14 cells and were infected with LCMV-Armstrong. 30 days after LCMV infection, mice were infected with 1 × 10^5^ colony forming units of Vir LM. Analysis was performed 1, 5, 20, or 48 h following Vir LM infection. Representative histograms of bystander IFN-γ production by gated P14 cells at the indicated time following Vir LM infection. Representative data from one of two independent experiments.

Figure S2 Bystander memory CD8 T cell responses decrease with time after initial antigen encounter in outbred mice. NIH Swiss mice were infected with LCMV-Armstrong. Either 30 days (earlyM) or >8 months (lateM) after LCMV infection, mice were infected with Vir *Listeria monocytogenes* (LM). Analysis was performed 20 h following Vir LM infection. **(A)** Representative histograms of bystander IFN-γ production by earlyM (left) or lateM (right) endogenous CD11a^hi^/CD8α^lo^ Ag-experienced cells (top), or endogenous CD11a^lo^/CD8α^hi^ naïve cells (bottom). **(B)** Summary graphs of the percentage of earlyM and lateM endogenous Ag-experienced cells producing IFN-γ (left) and IFN-γ MFI (right) 20 h after Vir LM infection. *n* = 7–8 mice/group. Representative data from one of two independent experiments. Bars represent mean. Unpaired *t*-test; ***p* < 0.01.

Figure S3 Bystander-mediated protection against unrelated *Listeria monocytogenes* (LM) infection is not influenced by time after initial antigen (Ag) encounter or Ag-encounter history. **(A)** Experimental design. IFN-γ knockout mice either received or did not receive adoptive transfer of 500,000 1° earlyM or lateM, or 3° earlyM P14 cells and were infected with Vir LM. 2 days after infection, spleens were harvested and bacterial colony forming units were enumerated. **(B)** Summary bar graph of LM colonies detected in spleens of IFN-γ knockout mice that received adoptive transfer of the indicated populations of 1° earlyM or lateM, or 3° earlyM P14 cells. *n* = 3 mice/group. Representative data from one of two independent experiments. Bars represent mean ± SEM. ANOVA with Bonferroni posttest; NS, not significant, **p* < 0.05.
