# Supplementary figures and images for: Time and Antigen-Stimulation History Influence Memory CD8 T Cell Bystander Responses
Source: Front Immunol. 2017 Jun 8;8:634. doi: 10.3389/fimmu.2017.00634 (PMC5462920; doi:10.3389/fimmu.2017.00634)

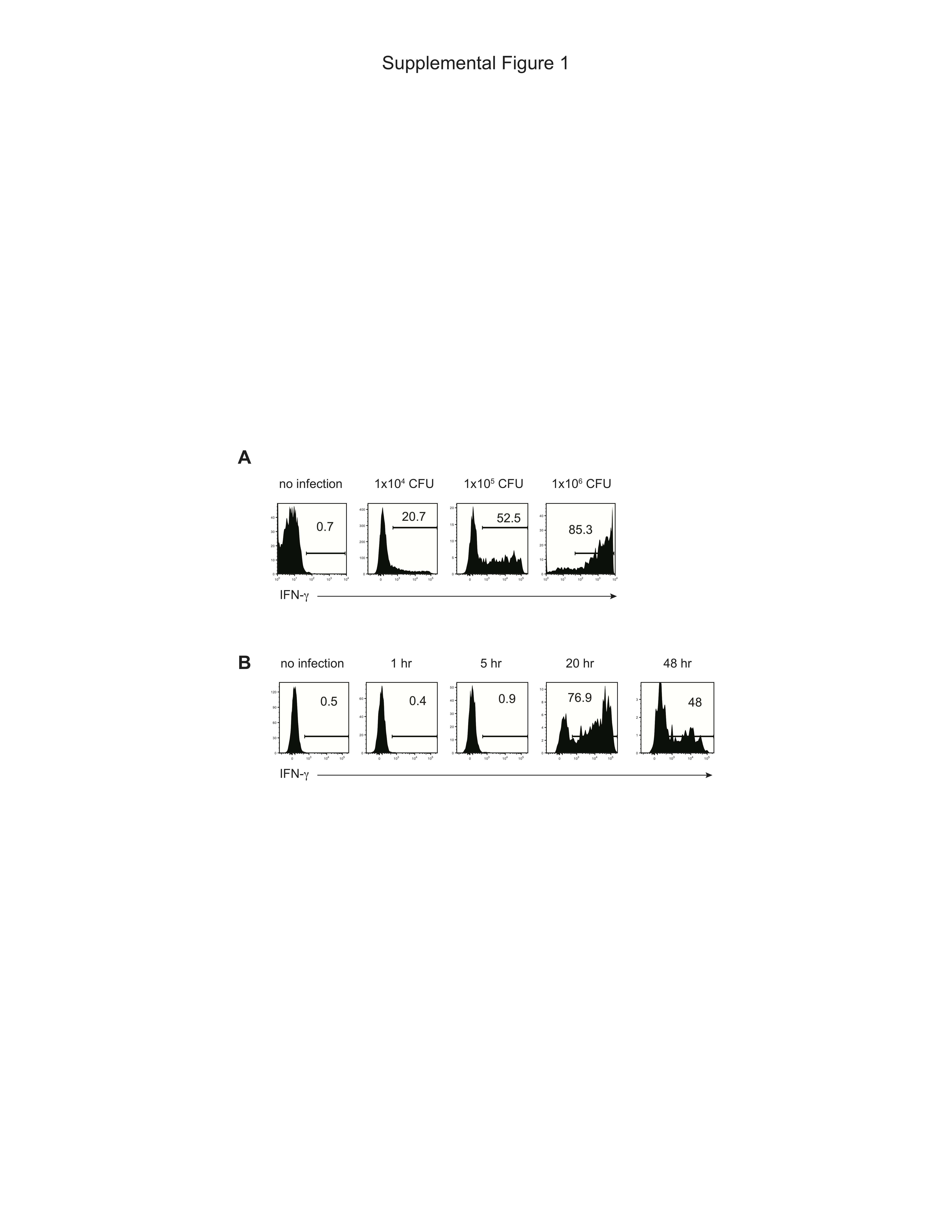

Supplement: Figure S1 — Bystander memory CD8 T cell responses occur in a dose-dependent manner and are robust 20 h after infection. (A) Mice received adoptive transfer of naïve P14 cells and were infected with LCMV-Armstrong. 200 days after LCMV infection, mice were infected with 1 × 10^4^, 1 × 10^5^, or 1 × 10^6^ colony forming units of Vir Listeria monocytogenes (LM). Analysis was performed 20 h following Vir LM infection. Representative histograms of bystander IFN-γ production by gated P14 cells following the indicated infectious dose of Vir LM. Representative data from one of two independent experiments. (B) Mice received adoptive transfer of naïve P14 cells and were infected with LCMV-Armstrong. 30 days after LCMV infection, mice were infected with 1 × 10^5^ colony forming units of Vir LM. Analysis was performed 1, 5, 20, or 48 h following Vir LM infection. Representative histograms of bystander IFN-γ production by gated P14 cells at the indicated time following Vir LM infection. Representative data from one of two independent experiments. [file image_1.tif]

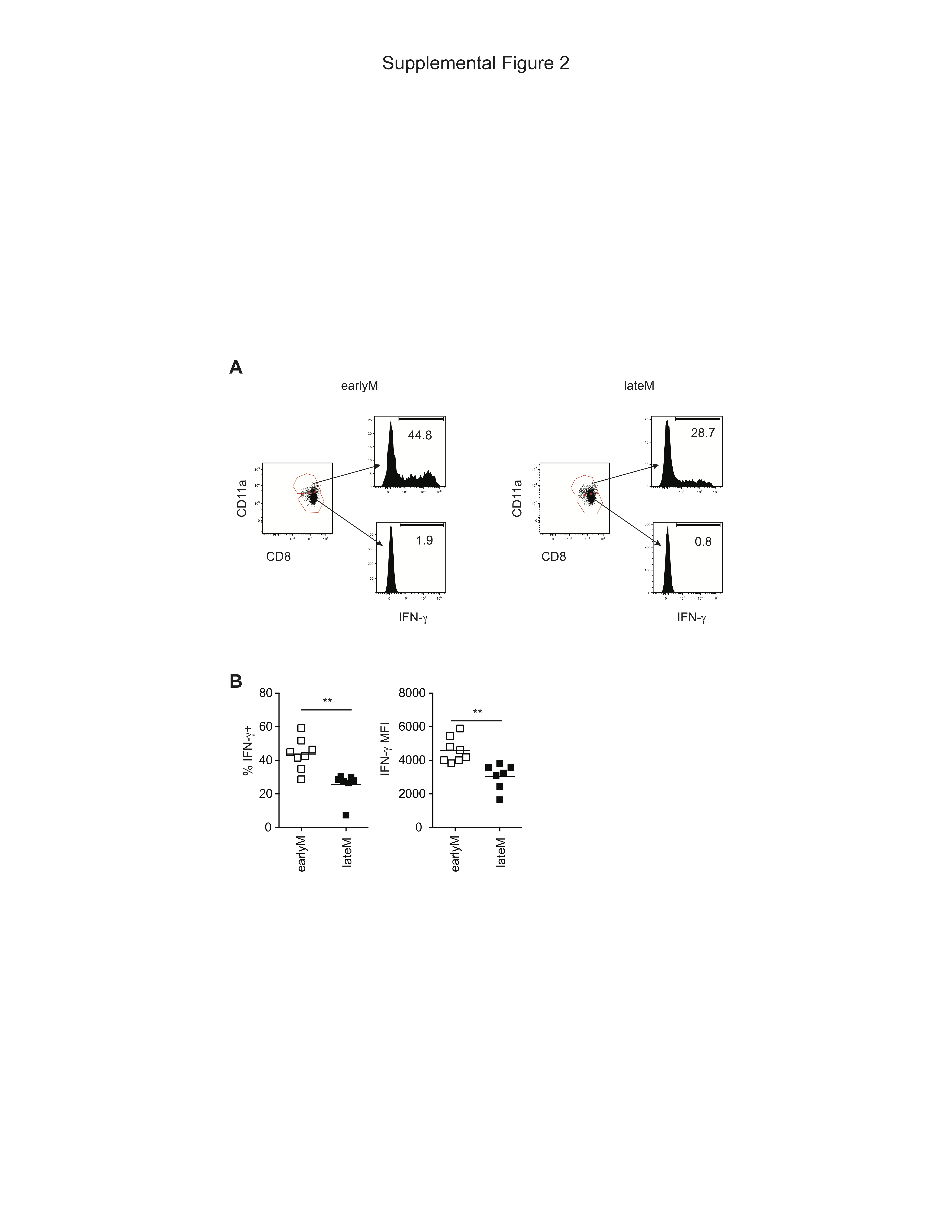

Supplement: Figure S2 — Bystander memory CD8 T cell responses decrease with time after initial antigen encounter in outbred mice. NIH Swiss mice were infected with LCMV-Armstrong. Either 30 days (earlyM) or >8 months (lateM) after LCMV infection, mice were infected with Vir Listeria monocytogenes (LM). Analysis was performed 20 h following Vir LM infection. (A) Representative histograms of bystander IFN-γ production by earlyM (left) or lateM (right) endogenous CD11ahi/CD8αlo Ag-experienced cells (top), or endogenous CD11alo/CD8αhi naïve cells (bottom). (B) Summary graphs of the percentage of earlyM and lateM endogenous Ag-experienced cells producing IFN-γ (left) and IFN-γ MFI (right) 20 h after Vir LM infection. n = 7–8 mice/group. Representative data from one of two independent experiments. Bars represent mean. Unpaired t-test; **p < 0.01. [file image_2.tif]

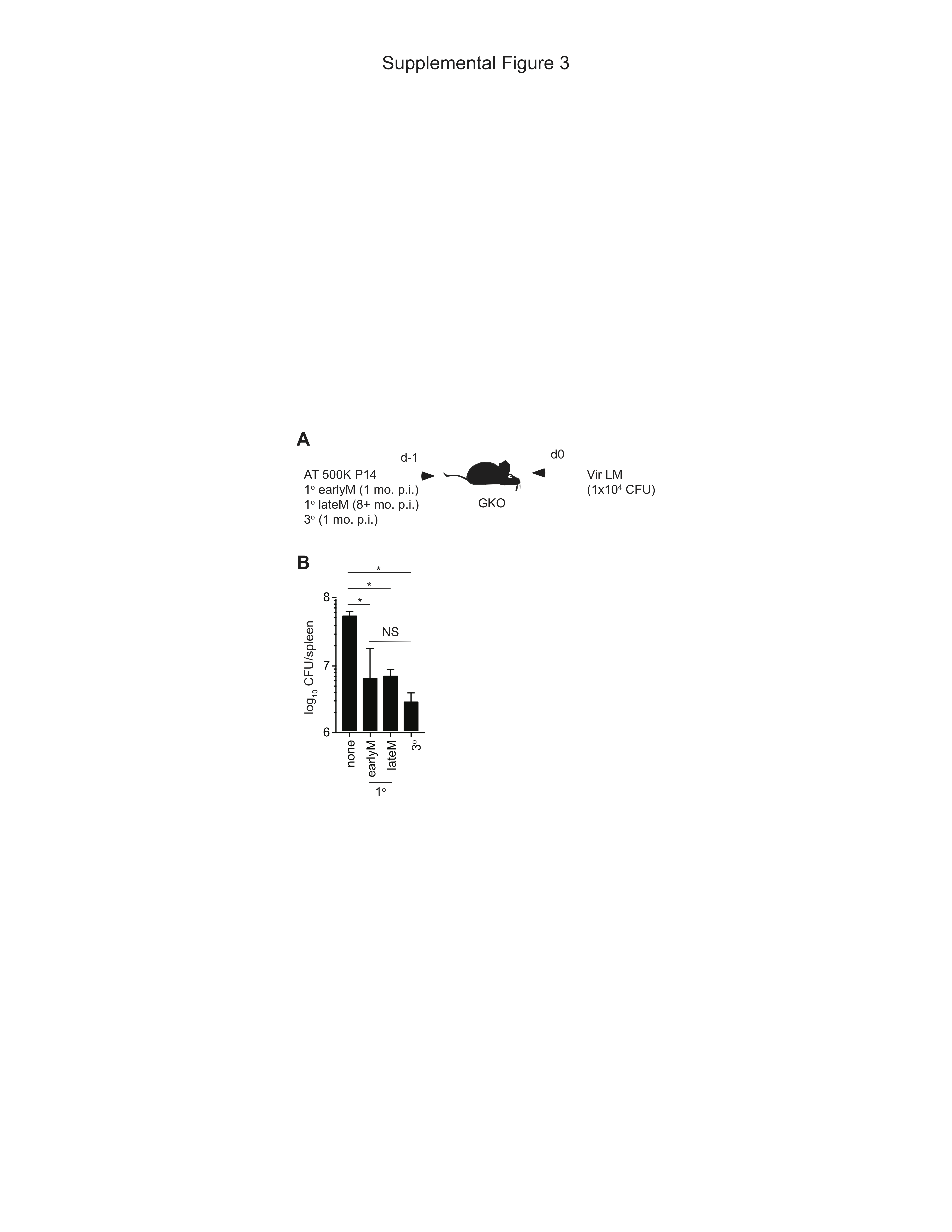

Supplement: Figure S3 — Bystander-mediated protection against unrelated Listeria monocytogenes (LM) infection is not influenced by time after initial antigen (Ag) encounter or Ag-encounter history. (A) Experimental design. IFN-γ knockout mice either received or did not receive adoptive transfer of 500,000 1° earlyM or lateM, or 3° earlyM P14 cells and were infected with Vir LM. 2 days after infection, spleens were harvested and bacterial colony forming units were enumerated. (B) Summary bar graph of LM colonies detected in spleens of IFN-γ knockout mice that received adoptive transfer of the indicated populations of 1° earlyM or lateM, or 3° earlyM P14 cells. n = 3 mice/group. Representative data from one of two independent experiments. Bars represent mean ± SEM. ANOVA with Bonferroni posttest; NS, not significant, *p < 0.05. [file image_3.tif]
